# Sex Differences in Diagnosis and Clinical Phenotypes of Chinese Children with Autism Spectrum Disorder

**DOI:** 10.1007/s12264-017-0102-9

**Published:** 2017-02-25

**Authors:** Shihuan Wang, Hongzhu Deng, Cong You, Kaiyun Chen, Jianying Li, Chun Tang, Chaoqun Ceng, Yuanyuan Zou, Xiaobing Zou

**Affiliations:** Child Developmental and Behavioral Center, Third Affiliated Hospital of Sun Yat-sen University, Guangzhou, 510630 China

**Keywords:** Autism spectrum disorder, Sex differences, Diagnosis

## Abstract

The aim of this study was to explore the differences between boys and girls in the diagnosis and clinical phenotypes of autism spectrum disorder (ASD) in China's mainland. Children diagnosed with ASD (*n* = 1064, 228 females) were retrospectively included in the analysis. All children were assessed using the Autism Diagnostic Interview-Revised (ADI-R) and Autism Diagnostic Observation Schedule (ADOS). The results showed that girls scored significantly higher in ADI-R socio-emotional reciprocity than boys, and also scored lower in ADI-R and ADOS restricted and repetitive behaviors (RRBs). Meanwhile, the proportions of girls who satisfied the diagnostic cut-off scores in the ADI-R RRBs domain were lower than in boys (*P* < 0.05). Our results indicated that girls with ASD show greater socio-emotional reciprocity than boys. Girls also tended to show fewer RRBs than boys, and the type of RRBs in girls differ from those in boys. The ADI-R was found to be less sensitive in girls, particularly for assessment in the RRBs domain.

## Introduction

Autism spectrum disorder (ASD) is a set of heterogeneous neurodevelopmental disorders characterized by developmental delays in social communication and restricted and repetitive behaviors (RRBs) [1]. Based on the most recent epidemiological surveys, the global prevalence of ASD is estimated to be 1%–2% [2, 3]. Males are disproportionately represented at ~4:1 [4, 5]. While epidemiological studies have confirmed the male dominance in ASD, the reason for this is unclear. The original description, diagnostic criteria, and clinical data for ASD were based almost solely on males, with relatively few studies focusing on females. Several studies have reported that females with ASD might exhibit behaviors, cognitive functioning, neuroanatomy, and gene expression patterns different from males [6–8]. However, the characterization of ASD in females is far from complete.

Few studies have explored sex differences within the core clinical phenotypes in children with ASD, and the results are inconsistent. Some studies have reported greater stereotypical play and RRBs in males with ASD. Bölte *et al.* found that males exhibit more RRBs than females in adult high-functioning autism as assessed using the Autism Diagnostic Observation Schedule (ADOS) [9]. Hattier *et al.* also reported a higher frequency of RRBs in adult males regardless of age range as assessed using the Stereotypies subscale of the Diagnostic Assessment for the Severely Handicapped-II [10]. However, some investigators have found no such sex differences in the RRBs domain [11, 12]. In the social communication domain, Frazier *et al.* recently reported that females with ASD (age range, 4–18 years) have greater social communication impairment than males [13]. Hiller *et al.* reported that girls with ASD are more likely to integrate non-verbal and verbal behaviors, maintain reciprocal conversation, and be able to initiate friendships [14]. In contrast, other studies have found no sex differences in early social-communication skills [15]. Collectively, these studies suggest potential differences in the symptoms of ASD between males and females. However, a clear and consistent picture of the clinical phenotypes of ASD in females has not yet emerged. This may be due to variability in the age of patients, sample sizes, diagnostic criteria, and assessment tools used in previous studies.

Females have been reported to be more likely to experience a lack of diagnosis, delay in diagnosis, and misdiagnosis. Goin-Kochel *et al.* reported that girls were diagnosed later for Asperger’s disorder (average 8.9 *vs.* 7.0 years) and pervasive developmental disorder-not otherwise specified (average 5.1 *vs.* 3.9 years), when compared with boys [16]. Koenig and Tsatsanis highlighted that sex differences at the time of presentation have not been sufficiently addressed in validation studies of the key diagnostic instruments, such as the ADI-R and ADOS [17]. There is a paucity of research addressing the validity of diagnostic criteria, particularly in females. In addition, symptom criteria or assessment items may be biased, raising doubts about the criteria and content validity of the ADI-R and ADOS diagnostic algorithms, especially in relation to females.

Few studies have been conducted on sex differences in core clinical phenotypes in children with ASD, specifically in Asian populations. Early abnormal developmental differences between boys and girls with ASD remain unknown. The primary objective of the present study was to explore sex differences in the domains of social communication and RRBs in children with ASD in a large sample from an Asian community. The second objective was to retrospectively analyze the differences in early abnormal development between boys and girls with ASD based on the ADI-R. A third objective was to further explore the differences in diagnostic cut-off scores for ADI-R and ADOS between boys and girls with ASD.

## Methods

### Participants

The sample retrospectively included 1064 individuals (228 girls and 836 boys). These children were diagnosed with ASD in a single-center clinic—The Child Developmental & Behavioral Center in the Third Affiliated Hospital of Sun Yat-sen University, Guangzhou—between June 2013 and October 2015. The participants selected were 24–83 months old. Inclusion criteria: children who fulfilled the ASD diagnostic criteria based on the Diagnostic and Statistical Manual of Mental Disorders, 4th Edition, Text Revision (DSM-IV-TR) [18]. Exclusion criteria: children with mental retardation, idiopathic language retardation, or schizophrenia. There were no gender differences in the exclusion samples (11 girls and 47 boys).

### Diagnostic Assessment

#### The Autism Diagnostic Interview-Revised (ADI-R)

The ADI-R [19] is a semi-structured parent/caregiver interview designed to assess and quantify the developmental history of autism-specific behaviors. It contains 93 items, including development of early childhood, language development, communication functioning, social reciprocity, play, and RRBs. The ADI-R diagnostic items constitute 4 domains: social reciprocity (A: cut-off ≥10), communication (B: cut-off ≥8 for verbal and ≥7 for non-verbal individuals), RRBs (C: cut-off ≥3), and abnormal development before 36 months (D: cut-off ≥1). Verbal children were defined as those who have spontaneous, echoed, or stereotyped language, which on a daily basis, involves phrases of three words or more [19]. The cut-off scores were defined as satisfying the autism diagnostic criteria. The social reciprocity domain (A) consists of non-verbal behaviors to regulate social interaction (A1), developing peer relationships (A2), sharing enjoyment (A3), and socio-emotional reciprocity (A4). The communication domain (B) consists of gesture communication (B1), conversation (B2, only for verbal individuals), repetitive speech (B3, only for verbal individuals), and play (B4). The RRBs domain (C) consists of unusual preoccupation, circumscribed interest, verbal rituals, compulsions/rituals, hand and finger mannerisms, stereotyped body movements, repetitive use of objects/interest in parts of objects, and unusual sensory interest. Abnormal development before 36 months (D) consists of age when parents first noticed developmental abnormalities, age when developmental abnormalities probably first manifested in interviewer’s judgment, age of first single words, and age of first phrases. In the ADI-R items, word speech delay is defined as the age at first single words >24 months, and phrase speech delay is defined as the age at first phrase >33 months.

#### The Autism Diagnostic Observation Schedule (ADOS)

The ADOS is a standardized assessment tool for children with suspected ASD [20]. It involves a semi-structured interview with interspersed activities and tasks intended to elicit behaviors associated with ASD. It covers communication, social reciprocity, play/imagination, and RRBs. Depending on the child’s language level, verbal children received module 2 assessment, while non-verbal children received module 1 assessment. The cut-off scores for satisfying the autism diagnostic criteria were defined in the domains of social reciprocity (A) and communication (B). For module 1, the cut-off for autism was A + B ≥ 12, with A ≥ 7 and B ≥ 4. For module 2, the cut-off for autism was A + B ≥ 12, with A ≥ 6 and B ≥ 5.

### Statistical Analyses

Data were analyzed using the Statistical Package for Social Sciences (version 20.0; SPSS Inc., Chicago, IL). The differences in baseline characteristics between boys and girls with ASD were examined using χ^2^-test. The scores in the social communication domain were normally distributed, while the scores for different types of RRBs were skewed. Sex differences in the scores for the social communication domain and early abnormal development were tested using Analysis of Covariance, with sex as the fixed factor and age as the covariate. The differences in the scores for different types of RRBs between boys and girls with ASD were determined using Mann–Whitney U tests. The differences in cut-off scores with respect to social reciprocity, communication, and RRBs between boys and girls with ASD were examined using χ^2^-test. *P* < 0.05 was considered statistically significant. Effect Size (ES) was used to estimate the sex effect.

## Results

### Demographic Characteristics

The baseline demographic characteristics are listed in Table 1. There were no statistically significant age differences between boys and girls for both verbal and non-verbal children. Word and phrase speech delay was more frequently reported in girls than in boys (χ^2^ = 21.82, 7.67; *P* < 0.05; ES = 0.14, 0.09). While most children were diagnosed with autism, only 7.46% of girls and 7.66% of boys were diagnosed with Asperger’s disorder. There were no sex differences in the distribution of diagnoses.

**Table 1:** Baseline demographic characteristics of children with ASD.

	Girls with ASD	Boys with ASD	Effect size	t/χ^2^	*P*
	Mean (SD)	Mean (SD)			
*n*	228	836	0.03	0.84	0.361
Verbal (%)	134 (58.77%)	463 (55.38%)			
Non-verbal (%)	94 (41.23%)	373 (44.62%)			
*Age in months*					
Verbal	52.04 (17.86)	55.07 (16.32)	0.18	1.85	0.065
Non-verbal	35.49 (10.79)	36.37 (10.84)	0.06	0.71	0.48
Word speech delay (*n*, %)	155 (67.98)	423 (50.60)	0.14	21.82	<0.001
Phrase speech delay (*n*, %)	103 (45.18)	294 (35.17)	0.09	7.67	0.006
*Diagnosis* (*n*, %)					
Autism	204 (89.47)	761 (91.03)	0.06	0.51	0.474
Asperger’s disorder	17 (7.46)	64 (7.66)	0.01	0.01	0.92
PDD-NOS	7 (3.07)	11 (1.31)	0.18	3.32	0.069

ASD, autism spectrum disorder; PDD-NOS, pervasive developmental disorder-not otherwise specified; SD, standard deviation.

### Sex Differences in Social Reciprocity and Communication Domains

No significant between-group differences were found in total social reciprocity scores based on ADI-R and ADOS in verbal and non-verbal children (Table 2). However, detailed analysis of social reciprocity revealed that verbal and non-verbal girls with ASD scored higher in terms of ADI-R socio-emotional reciprocity than boys (*P* = 0.049, 0.001; ES = 0.22, 0.38).

**Table 2:** Descriptive statistics for social reciprocity domain in girls and boys with ASD.

Scores	Girls with ASD	Boys with ASD	Effect size	*F*	*P*
	Mean (SD)	Mean (SD)			
*Non-verbal behaviors to regulate social interaction (A1)*					
Verbal	3.20 (1.22)	3.04 (1.18)	0.13	1.69	0.194
Non-verbal	3.69 (1.25)	3.56 (1.30)	0.10	0.86	0.353
*Develop peer relationships (A2)*					
Verbal	4.13 (1.85)	4.13 (1.90)	0.00	1.17	0.279
Non-verbal	3.20 (1.60)	3.17 (1.55)	0.02	0.33	0.568
*Share enjoyment (A3)*					
Verbal	3.77 (1.48)	3.62 (1.52)	0.10	0.46	0.499
Non-verbal	4.59 (1.27)	4.60 (1.35)	0.01	0.01	0.907
*Socio-emotional reciprocity (A4)*					
Verbal	4.99 (1.98)	4.62 (1.60)	0.22	3.89	0.049
Non-verbal	6.35 (1.84)	5.70 (1.65)	0.38	11.08	0.001
*ADI-R social reciprocity domain (A)*					
Verbal	16.10 (4.83)	15.41 (4.02)	0.16	3.30	0.070
Non-verbal	17.83 (4.57)	17.03 (4.21)	0.19	3.12	0.078
*ADOS social reciprocity domain (A)*					
Verbal	8.93 (2.74)	8.80 (2.80)	0.04	0.13	0.715
Non-verbal	9.86 (2.33)	9.42 (2.68)	0.17	1.97	0.161

ADI-R Social reciprocity domain A = A1 + A2 + A3 + A4.

ASD, autism spectrum disorder; SD, standard deviation.

No sex-based differences were found in total verbal communication scores based on ADI-R and ADOS in verbal children (Table 3). However, verbal girls with ASD scored higher in ADI-R gesture communication than boys (*P* < 0.001; ES = 0.40), and non-verbal girls scored higher in the ADOS communication domain than boys (*P* = 0.006; ES = 0.32). In addition, verbal girls scored lower in ADI-R repetitive speech than boys (*P* = 0.003; ES = 0.29).

**Table 3:** Descriptive statistics for the communication domain in girls and boys with ASD.

Scores	Girls with ASD	Boys with ASD	Effect size	*F*	*P*
	Mean (SD)	Mean (SD)			
*Gesture communication (B1)*					
Verbal	4.12 (2.06)	3.27 (2.07)	0.40	14.54	<0.001
Non-verbal	5.78 (1.71)	5.62 (1.92)	0.08	0.52	0.473
*Conversation (B2)*					
Verbal	2.69 (1.38)	2.90 (1.44)	0.14	1.70	0.192
*Repetitive speech (B3)*					
Verbal	2.78 (1.01)	3.10 (1.16)	0.29	9.04	0.003
*Play (B4)*					
Verbal	3.87 (1.31)	4.03 (1.33)	0.12	1.42	0.233
Non-verbal	4.99 (1.07)	4.97 (1.01)	0.02	0.04	0.85
*ADI-R communication domain (B)*					
Verbal	13.46 (3.44)	13.30 (3.63)	0.05	0.05	0.824
Non-verbal	10.77 (2.36)	10.59 (2.50)	0.07	0.39	0.533
*ADOS communication domain (B)*					
Verbal	5.87 (1.88)	5.50 (2.07)	0.18	2.32	0.128
Non-verbal	6.56 (1.58)	5.99 (1.85)	0.32	7.65	0.006

ADI-R communication domain: B (verbal) = B1 + B2 + B3 + B4; B (non-verbal) = B1 +B4.

ASD, autism spectrum disorder; SD, standard deviation.

### Sex Differences in RRBs Domain

Girls with ASD (3.59 ± 1.87) scored lower than boys (4.55 ± 2.06) in total RRBs based on the ADI-R (*F* = 39.03, *P* < 0.001; ES = 0.32), and girls with ASD (2.02 ± 1.47) also scored lower in RRBs than boys (2.30 ± 1.41) based on the ADOS (*F* = 7.73, *P* = 0.006; ES = 0.13). Based on the ADI-R, non-verbal girls with ASD scored higher than boys in hand and finger mannerisms and stereotyped body movements (*Z* = 2.13, 2.22; *P* = 0.033, 0.026). Conversely, non-verbal boys with ASD scored higher than girls in unusual preoccupation, repetitive use of objects, and interest in parts of objects (*Z* = 2.15, 7.95; all *P* < 0.05). In addition, verbal boys with ASD scored higher than girls in unusual preoccupation, circumscribed interest, verbal rituals, repetitive use of objects, and interest in parts of objects (*Z* = 2.83, 2.54, 2.98, 9.22; all *P* < 0.05) (Table 4).

**Table 4:** Descriptive statistics for repetitive stereotyped behaviors domain in girls and boys with ASD.

Scores	Girls (*n* = 228)			Boys (*n* = 836)			*Z*	*P*
	0	1	2	0	1	2		
*Unusual preoccupation*								
Verbal	90 (39.47%)	33 (14.47%)	11 (4.82%)	252 (30.14%)	137 (16.39%)	74 (8.85%)	2.83	0.005
Non-verbal	60 (26.32%)	28 (12.28%)	6 (2.63%)	200 (23.92%)	116 (13.88%)	57 (6.82%)	2.15	0.032
*Circumscribed interest*								
Verbal	68 (29.82%)	54 (23.68%)	12 (5.26%)	201 (24.04%)	158 (18.90%)	104 (12.44%)	2.54	0.011
Non-verbal	64 (28.07%)	25 (10.96%)	5 (2.19%)	285 (34.09%)	62 (7.42%)	26 (3.11%)	1.45	0.141
Verbal rituals*								
Verbal	91 (39.91%)	37 (16.23%)	6 (2.63%)	261 (31.22%)	126 (15.07%)	76 (9.09%)	2.98	0.003
*Compulsions/rituals*								
Verbal	58 (25.44%)	55 (24.12%)	21 (9.21%)	257 (30.74%)	110 (13.16%)	96 (11.48%)	1.45	0.148
Non-verbal	54 (23.68%)	31 (13.60%)	9 (3.95%)	247 (29.55%)	69 (8.25%)	57 (6.82%)	0.97	0.331
*Hand and finger mannerisms*								
Verbal	87 (38.16%)	36 (15.79%)	11 (4.82%)	325 (38.88%)	83 (9.93%)	55 (6.58%)	0.77	0.442
Non-verbal	51 (22.37%)	28 (12.28%)	15 (6.58%)	248 (29.67%)	80 (9.57%)	45 (5.38%)	2.13	0.033
*Stereotyped body movements*								
Verbal	81 (35.53%)	39 (17.11%)	14 (6.14%)	256 (30.62%)	114 (13.64%)	93 (11.12%)	1.67	0.096
Non-verbal	34 (14.91%)	40 (17.54%)	20 (8.77%)	201 (24.04%)	92 (11.00%)	81 (9.69%)	2.22	0.026
*Repetitive use of objects/interest in parts of objects*								
Verbal	72 (31.58%)	50 (21.93%)	12 (5.26%)	73 (8.73%)	206 (24.64%)	184 (22.01%)	9.22	<0.001
Non-verbal	37 (16.23%)	43 (18.86%)	14 (6.14%)	33 (3.95%)	145 (17.34%)	195 (23.33%)	7.95	<0.001
*Unusual sensory interest*								
Verbal	58 (25.44%)	67 (29.39%)	9 (3.95%)	213 (25.48%)	254 (30.38%)	26 (3.11%)	0.45	0.881
Non-verbal	26 (14.40%)	48 (21.05%)	20 (8.77%)	114 (13.64%)	219 (26.20%)	40 (4.78%)	1.68	0.093

*P < 0.05; all comparisons between boys and girls with ASD (autism spectrum disorder).

Scores for different types of RRBs are ranked data; differences in skewed scores between boys and girls with ASD compared using Mann–Whitney U tests.

### Sex Differences in Early Abnormal Development

Based on the ADI-R, the age when parents first noticed developmental abnormalities in both verbal and non-verbal girls was later than in boys (*F* = 34.06, 51.09; all *P* < 0.001; ES = 0.45, 0.54). Meanwhile, the age when developmental abnormalities probably first manifested in the interviewer’s judgment in both verbal and non-verbal girls was also later than in boys (*F* = 114.27, 115.56; *P* < 0.001; ES = 0.44, 0.56). Furthermore, the age at which single words and phrases were first spoken by verbal girls was higher than that of boys (*F* = 6.94, 8.16; *P* = 0.009, 0.004; ES = 0.25, 0.26) (Table 5).

**Table 5:** Comparison of early abnormal development in girls and boys with ASD.

Age (months)	Girls with ASD	Boys with ASD	Effect size	*F*	*P*
	Mean (SD)	Mean (SD)			
*Age when parents first noticed developmental abnormalities*					
Verbal	34.71 (12.80)	29.73 (10.61)	0.45	34.06	<0.001
Non-verbal	27.27 (11.03)	21.94 (6.69)	0.54	51.09	<0.001
*Age when developmental abnormalities probably first manifest in interviewer’s judgment*					
Verbal	32.21 (9.72)	24.50 (7.32)	0.44	114.27	<0.001
Non-verbal	27.45 (8.88)	20.26 (5.85)	0.56	115.56	<0.001
*Age of first single words*					
Verbal	23.81 (8.11)	21.79 (7.85)	0.25	6.94	0.009
*Age of first phrases*					
Verbal	31.25 (9.51)	28.98 (8.29)	0.26	8.16	0.004

ASD, autism spectrum disorder; SD, standard deviation.

### Sex Differences in Diagnostic Cut-off Scores

The differences in diagnostic cut-off scores in boys and girls with ASD are summarized in Table 6. A lower proportion of verbal girls with ASD satisfied the cut-off scores for ASD relative to boys (89.85%) in the ADI-R repetitive stereotyped behaviors domain (χ^2^ = 20.53, *P* < 0.001, ES = 0.19). A lower proportion of non-verbal girls (73.40%) satisfied the cut-off scores for ASD relative to boys (84.72%) in the same domain (χ^2^ = 6.64, *P* = 0.010, ES = 0.12).

**Table 6:** Descriptive statistics for cut-off scores in girls and boys with ASD.

	Girls with ASD	Boys with ASD	Effect size	χ^2^	*P*
	Satisfied cut-off scores (*n*)	Satisfied cut-off scores (*n*)			
*ADI-R social reciprocity (A)*					
Verbal	123 (91.79%)	447 (96.54%)	0.10	5.44	0.020
Non-verbal	88 (93.62%)	361 (96.78%)	0.07	2.03	0.154
*ADI-R communication (B)*					
Verbal	127 (94.78%)	450 (97.19%)	0.06	1.87	0.171
Non-verbal	91 (96.81%)	351 (94.10%)	0.05	1.09	0.297
*ADI-R RRBs (C)*					
Verbal	100 (74.63%)	416 (89.85%)	0.19	20.53	<0.001
Non-verbal	69 (73.40%)	316 (84.72%)	0.12	6.64	0.010
*ADI-R abnormal development before 36* *months (D)*					
Verbal	130 (97.01%)	458 (98.92%)	0.06	2.54	0.111
Non-verbal	93 (98.93%)	370 (99.20%)	0.01	0.06	0.807
*ADOS communication (B)*					
Verbal	128 (95.52%)	426 (92.01%)	0.06	1.92	0.166
Non-verbal	93 (98.94%)	370 (99.20%)	0.01	0.06	0.807
*ADOS social reciprocity (A)*					
Verbal	131 (97.76%)	452 (97.62%)	0.00	0.01	0.926
Non-verbal	94 (100.00%)	366 (98.12%)	0.06	1.79	0.181
*ADOS communication* *+* *social reciprocity (A* *+* *B)*					
Verbal	130 (97.01%)	432 (93.30%)	0.07	2.59	0.107
Non-verbal	94 (100.00%)	364 (97.59%)	0.07	2.31	0.128

## Discussion

### Sex Differences in Core Clinical Phenotypes in Children with ASD

An important finding emerging from our study is the strong suggestion that both verbal and non-verbal girls with ASD have greater socio-emotional reciprocity impairment than boys, while non-verbal girls show more serious communication impairment than boys. Socio-emotional reciprocity includes use of the body to communicate, offering comfort, quality of expression of social interest, appropriate facial expressions, and appropriateness of social response. Holtmann *et al.* examined sex differences using the ADI-R and ADOS for participants with high-functioning autism matched for age (range, 5–20 years), and found that females have greater impairment in playing with the peer group and social problems as per the reports of parents based on ADI-R [21]. A recent study by Howe *et al.* revealed that verbal girls with ASD show greater impairment of social communication than males, based on the ADOS [22]. A possible explanation for this could be related to lower cognitive function in girls with ASD. Previous studies have suggested that girls with ASD have lower cognitive ability than boys [23]; Frazier also pointed out that females with a lower IQ have greater communication impairment [13]. The results of the present study suggest that girls with ASD exhibit a clinical phenotype different from that in boys.

To date, very few studies have documented differences in RRBs between girls and boys. In the present study, we found that girls with ASD showed fewer RRBs than boys, using both the ADI-R and ADOS. We also found that girls with ASD exhibited more stereotyped body movements (e.g. repetitive circling and jumping up and down) and hand and finger mannerisms (mechanical play with the hand) than boys, while boys exhibited more unusual preoccupations (e.g. with metal objects, lights, and traffic signs), verbal rituals (e.g. questioning knowingly and forcing others to speak), repetitive use of objects, and interest in parts of objects (e.g. playing with wheels and turning the lights on and off). In addition, boys with ASD exhibited more repetitive speech than girls. These results suggest that girls with ASD show different types of RRBs than boys, and that girls more commonly develop special repetitive stereotyped behaviors.

Girls with ASD are more likely to mask atypical interest, and this would not be considered an RRB in girls. For example, parents may report that their daughter likes to play with dolls. However, when probed about exactly how she ‘played’, it could become apparent that every session involved repeated brushing of hair, with little flexibility or imagination. This condition can be misinterpreted as an imaginative game for girls, rather than as an RRB [24]. Moreover, some special characteristics of RRBs in girls were absent from the diagnostic algorithms. For example, ASD girls often carry the same books when going outside, which may also be considered an RRB, but this is not included among the diagnostic criteria in the ADI-R [25]. In addition, some activities in boys are more likely to be considered RRBs. For example, parents may report that their son likes to play with trains or dinosaurs. While this may be considered a “special interest”, on further inquiry it may be a little stronger without affecting other interests [26]. Consequently, clinicians should carefully look for RRBs in ASD children to identify those common to both boys and girls. The notion that girls show fewer RRBs may be a “protective” factor for girls that in turn makes a formal diagnosis of ASD more difficult. Szatmari *et al.* suggested that this “protective” mechanism may have an underlying genetic component, consistent with the gene-threshold model for girls with ASD [27]. This model assumes that the threshold for ASD in females is higher than in males [28]. In other words, females require a greater genetic load to manifest autistic behaviors. As a result, once females are formally diagnosed, their cognitive function and behavioral characteristics tend to be more severe than in males.

### Sex Differences in Identification and Diagnosis in Children with ASD

Our results revealed that the age when parents first noticed developmental abnormalities and the age when developmental abnormalities probably first manifested in the interviewer’s judgment in girls were later than in boys. Furthermore, the age at which single words/phrases were first spoken was also later in girls than in boys. Collectively, the results suggest that early abnormal development and behavioral characteristics for girls are not as easy to identify and are liable to be missed by both parents and evaluators. This may lead to delayed diagnosis of ASD in girls. Shattuck *et al.* reported that the age at which the diagnosis of ASD is made in girls is significantly later than in boys (average 6.1 *vs.* 5.6 years) [29]. Previous studies have reported no obvious sex differences in core symptoms after controlling for age and IQ. However, girls with ASD tend to show more emotional problems, attention deficit, and thought problems [14]. This suggests that girls are diagnosed only when they exhibit more behavioral problems. One possible explanation for this difference is that boys are comparatively more likely to exhibit hyperactivity and repetitive use of objects, and exhibit interest in parts of objects to trigger detection and identification by parents or clinicians. In contrast, the characteristic behaviors in ASD girls are not always as overt and thus are liable to be missed. Clinical symptoms in high-functioning autistic girls (e.g. those exhibiting fewer RRBs) are particularly prone to be missed or misdiagnosed.

We also revealed that the proportion of both verbal and non-verbal girls who satisfied the cut-off scores in the RRBs domain was lower than in boys when assessed using the ADI-R. The ADI-R may be less sensitive for diagnosing ASD in girls, particularly in the RRBs domain. Girls with ASD may be under-identified due to RRBs not satisfying the cut-off scores for diagnosis. Wilson *et al.* noted that sex affects the diagnosis and evaluation of ASD, suggesting that females and males demonstrate distinct clinical phenotypes [26]. As such, sex differences need to be incorporated into the current diagnostic tools. This viewpoint has been articulated by several clinicians. There is therefore a call for tailoring the current diagnostic and assessment tools to address sex differences, in order to improve the diagnostic rate of ASD in girls.

## Conclusions

Our findings suggest that girls with ASD show greater socio-emotional reciprocity, and non-verbal girls suffer more communication impairment than boys. Girls tend to show fewer RRBs than boys, and the types of RRBs for girls may be different from those for boys. Early abnormal development and behavioral characteristics in girls are not easy to recognize. In addition, the ADI-R is less sensitive for girls, particularly assessment in the RRBs domain. Clarifying sex differences in diagnosis and clinical phenotype will assist in answering the question of why fewer girls are diagnosed with ASD than boys, and may provide clinical guidance for early screening, diagnosis, and intervention.
